# Autophagy contributes to the chemo-resistance of non-small cell lung cancer in hypoxic conditions

**DOI:** 10.1186/s12931-015-0285-4

**Published:** 2015-11-09

**Authors:** Jin Gu Lee, Ju Hye Shin, Hyo Sup Shim, Chang Young Lee, Dae Joon Kim, Young Sam Kim, Kyung Young Chung

**Affiliations:** Department of Thoracic and Cardiovascular Surgery, Yonsei University College of Medicine, Seoul, South Korea; Department of Internal Medicine, Yonsei University College of Medicine, Seoul, South Korea; Department of Pathology, Yonsei University College of Medicine, Seoul, South Korea; 50 Yonsei-ro, Seodaemun-gu, Seoul 120-752 South Korea

**Keywords:** Autophagy, Chemotherapy, Hypoxia, Lung cancer, Resistance

## Abstract

**Background:**

The development of chemo-resistance in non-small lung cancer is a major obstacle in treating patients. Hypoxia is a commonly faced microenvironment in solid tumor and suggested to be related to both autophagy and chemo-resistance.

**Methods:**

In this study, we investigated the role of hypoxia-induced autophagy in acquiring chemo-resistance in both cancer cell (A549) and human cancer tissue

**Results:**

Hypoxic exposure (1 % O2) of A549 cell stimulated autophagic induction in cancer cells, shown by increase of LC3BI to LC3BII conversion and decrease of p62/sequestosome1 in Western blot, increased GFP-LC puncta in confocal microscopy, and increased number of double-membrane autophagic vacuoles in electron micrographs. Hypoxic exposure also induced resistance of cancer cells to cisplatin, and LC3B siRNA restored the sensitivity of cancer cells to chemotherapy. Furthermore, Human lung cancer tissues that experienced chemotherapy showed increase of LC3BI to LC3BII conversion and decrease of p62/sequestosome1 compared with chemo-naïve cancer tissue in Western blot.

**Conclusion:**

Autophagy may play an important role in acquiring resistance to chemotherapy in lung cancer and hypoxia related pathway seems to be involved in autophagy induction.

## Introduction

Lung cancer is a common cause of cancer-related death worldwide [1]. Non-small cell lung cancer (NSCLC) composes more than 80 % of lung cancer and chemotherapy is a major treatment option for NSCLC patients. However, even the best regimens have an overall response rate of only 30–50 %. This poor response to chemotherapy leads to the treatment failure in lung cancer.

Autophagy is considered to have dual functions in cancer as both a tumor suppressor and a protector of tumor cell survival [2, 3]. Before a tumor develops, autophagy suppresses the tumor; however, once the tumor begins to develop, autophagy supports the tumor in harsh microenvironments. Some studies found that autophagy is up-regulated in response to cancer treatments, and protects tumor cells by contributing to the development of treatment resistance. This observation implies that pharmacological modulation of autophagy can be a therapeutic target for tumor cells [2–6].

Tumor cells often face hasher microenvironments than normal cells. Hypoxia occurs in 90 % of solid tumors because angiogenesis in tumor cannot supply rapidly growing tumor masses with enough oxygen [7–10]. Hypoxia also influences several cancer-related pathways including angiogenesis signaling, cell migration, energy metabolism, cell growth, and damage deoxyribonucleic acid (DNA) repair. Recent studies found relationships between hypoxia-related pathways and autophagy activation. These relationships include oxygen-dependent stabilization of hypoxia-inducible factor 1-alpha (HIF-1α) transcription factor, inhibition of the mammalian target of rapamycin (mTOR) kinase signaling pathway and activation of the unfolded protein response (UPR) [11–13]. HIF-1α is the main regulator of transcription during hypoxia. HIF-1α overexpression is frequently observed in tumors and is considered to contribute to tumor cell growth and survival by controlling both glycolysis and angiogenesis. However, the effect of autophagy in tumor cells under hypoxia is not fully understood.

We hypothesized that hypoxia-induced autophagy might contribute to the resistance of NSCLC cells to chemotherapeutic agents by decreasing their apoptotic potential and enhancing their survival. To test this hypothesis, we studied the effect of hypoxia on tumor cells treated with chemotherapeutic agents and analyzed the effects of autophagy. Furthermore, to support our hypothesis, we measured HIF-1α expression in tumor tissues and autophagy markers in tumor tissues obtained from NSCLC patients.

## Materials and methods

### Cell lines and cultures

The A549 human lung cancer cell line was from the Korean Cell Line Bank just before use and it wasn’t validated again. A549 cells were maintained as monolayers in RPMI 1640 medium (GIBCOBRL, Gaithersburg, MD, USA) containing 10 % fetal bovine serum, 100 U/ml penicillin (Thermo Scientific, Rockford, IL, USA) and 0.1 mg/ml streptomycin (Thermo Scientific,) at 37.0 °C under 5 % CO_2_.

### Reagents

The chemotherapeutic agent cisplatin (Sigma, St. Louis, MO, USA) was dissolved in RPMI 1640 medium to produce a stock solution (1 mM) and added directly to media at indicated concentrations.

### Hypoxia treatment

For induction of hypoxic conditions, cells (1 × 104 cells/well) were seeded in 96-well flat-bottomed plates overnight, then incubated in hypoxia incubator (Forma Scientific, Ohio, USA) with 1 % O2.

### MTT colorimetric assays

To investigate the influence of hypoxia on cancer cell chemosensitivity, A549 cells were seeded in 96-well plates at 1 × 10^4^ cells/well and cultured in 1 % hypoxia or normoxia in medium containing cisplatin as indicated. Cell viability was examined with MTT assay kits (Sigma). Spectrophotometric absorbance was measured using a plate reader at 570 nm. All experiments were carried out in triplicate.

### Transient transfection and identification of autophagy

A549 cells were seeded (6.25 × 105 cells/well) in 6-well plates overnight, and a GFP-LC3 expressing plasmid was transiently transfected into cells using Lipofectamine LTX Reagent (Invitrogen, Carlsbad, CA, USA), according to the manufacturer’s instructions. After 24 h and verifying the expression of GFP-LC3, cells were subjected to hypoxia (1 % O_2_) for 8 h. After exposure, autophagy (GFP-LC3-positive dots) was counted (GFP-LC3-positive dots) under a confocal laser microscope, LSM700 (Carl Zeiss, Jena, Germany).

### Electron microscopy

Samples were fixed with 2 % glutaraldehyde-paraformaldehyde in 0.1 M phosphate buffer (PB), pH 7.4 for 12 h at 4 °C and washed three times for 30 min in 0.1 M PB. Samples were postfixed with 1 % OsO4 dissolved in 0.1 M PB for 2 h and dehydrated in an ascending gradual series (50–100 %) of ethanol and infiltrated with propylene oxide. Specimens were embedded using a Poly/Bed 812 kit (Polysciences, Warrington, PA, USA). After pure fresh resin embedment and polymerization at 60 °C in an electron microscope oven (TD-700, DOSAKA, Kyoto, Japan) for 24 h, 350-nm sections were cut and stained with toluidine blue for light microscopy and 70-nm thin sections were double stained with 7 % uranyl acetate and lead citrate for contrast staining. Sections were made with a LEICA Ultracut UCT Ultra-microtome (Leica Microsystems, Wetzlar, Germany). All thin sections were observed by TEM (JEM-1011, JEOL, Tokyo, Japan) at an acceleration voltage of 80 kV.

### Western blot

Expression of autophagy-related proteins was analyzed by Western blot. After indicated treatments, A549 cells were lysed in RIPA lysis buffer (Biosesang, Seongnam, Gyeonggi, KOREA) with 1 mM PMSF. Equal amounts of proteins were separated by SDS-PAGE and transferred to PVDF membranes (Millipore Corporation, Billerica, MA, USA). After blocking with 5 % nonfat milk, membranes were probed with anti-HIF-1α (BD Biosciences, San Jose, California, USA), anti-LC3 (Sigma), anti-p62C-terminus/SQSTM1 (Santa Cruz Biotechnology, Santa Cruz, CA, USA), anti-beclin-1 (Santa Cruz), or anti-β-actin (Sigma) and developed with ECL (Thermo Scientific, Rockford, IL, USA) reagent.

### NSCLC patients and samples

To determine overexpression of autophagy-related factors after chemotherapy, we selected five NSCLC patients (three adenocarcinoma and two squamous cell carcinoma) who had not yet been treated and five NSCLC patients (two adenocarcinoma and three squamous cell carcinoma) who received neoadjuvant treatment before operation as our study group. All patients underwent complete tumor resection and mediastinal lymph node dissection at the Department of Thoracic and Cardiovascular Surgery of Severance Hospital of Yonsei Medical University. The research was approved by the Institutional Review Board of Yonsei University, College of Medicine. All patients gave signed, informed consent. Tumor specimens and surrounding normal lung tissue (separated from tumors by 5 cm) were obtained at surgery, or selected by a pathologist, and stored. Specimens were divided into three groups: protein preparation, TEM and IHC.

### Immunohistochemisty for Hypoxia expression

Surgical specimens were immediately transferred from the operating room to the laboratory, fixed in 10 % formaldehyde for four days, and embedded in paraffin. Formalin-fixed and paraffin-embedded tissues were sectioned at 4 um and stained with antibody against HIF-1α (ab2185, 1:50, Abcam, Cambridge, UK), Carbonic anhydrase IX (CA IX) (ab15086, 1:300, Abcam, Cambridge, UK) and Glucose transporter 1 (GLUT1) (ab652, 1:100, Abcam, Cambridge, UK) using a Ventana automated immunostainer (Ventana Medical Systems, Tucson, AZ) according to the manufacturer’s protocol. Signals were detected using a DAB Map detection kit (Ventana Medical Systems) based on the labeled streptavidin-biotin method. The frequency of nuclear staining was evaluated without knowledge of clinical or pathologic status. Five fields (x200) were analyzed to determine the frequency of HIF-1α stained nuclei. At least 500 tumor cells in the five fields were counted. HIF-1α staining was evaluated using a semiquantitative grading system based on the number of tumor cells with completely dark stained nuclei within the tumor tissue (0: none, 1: less than 10 % positivity, 2: 10 % or greater positivity). Occasional cytoplasmic staining was ignored because active HIF-1α is located only in the nucleus [14]. Stained slides were graded independently by a single pathologist (HS Shim).

### Protein extraction and Western blotting of tumor tissues

Tumor tissues and adjacent tissues were homogenized in 1 ml RIPA buffer (50 mM Tris–HCl, pH 7.5, 0.1 % SDS, 2 mM EDTA, 150 mM NaCl, 1 % sodium deoxycholate, 1 % Triton X-100) and protease inhibitor cocktail. Homogenate was incubated for 20 min on ice and centrifuged at 14,000 rpm for 15 min at 4 °C. Supernatant was collected and the same volume of 5X SDS buffer was added. The mixture was boiled for 5 min and stored at −80 °C. Proteins were separated by sodium dodecy1 sulfate polyacrylamide gel electrophoresis (SDS-PAGE) on 8–16 % polyacrylamide gels and transferred to PVDF membranes (Millipore Corporation, Billerica, MA, USA), Membranes were blocked in 5 % nonfat milk in Tris-buffered saline containing 0.05 % Tween-20 (TBST) at room temperature for 1 h. Membranes were incubated with rabbit monoclonal antibody against LC3 (1:2000 diluted in TBST, Novus, USA) overnight at 4 °C and washed five times with TBST for 10 min at room temperature. Membranes were incubated with horseradish peroxidase-conjugated anti-rabbit secondary antibody (1:2000 dilution in TBST, Santa Cruz) at 37 °C for 1 h and washed five times with TBST for 10 min at room temperature. Membranes were treated with ECL (Thermo Scientific, Rockford, IL, USA) reagent and exposed to photographic film. Bands were quantified using densitometry and results were shown as relative expression of the protein from different samples compared to a control sample.

### Statistical analysis

MTT assays were analyzed using SoftMax Pro5.4 (Molecular Devices Corporation, Sunnyvale, CA, USA). All experiments were repeated at least three times. Data were expressed as mean ± standard error of the mean (SEM). Statistical analysis was performed using Student’s *t*-test or ANOVA (two-tailed). The criterion for statistical significance was *p* < 0.05.

## Results

### Hypoxia induces expression of HIF-1α in cancer cells

**Fig. 1:**
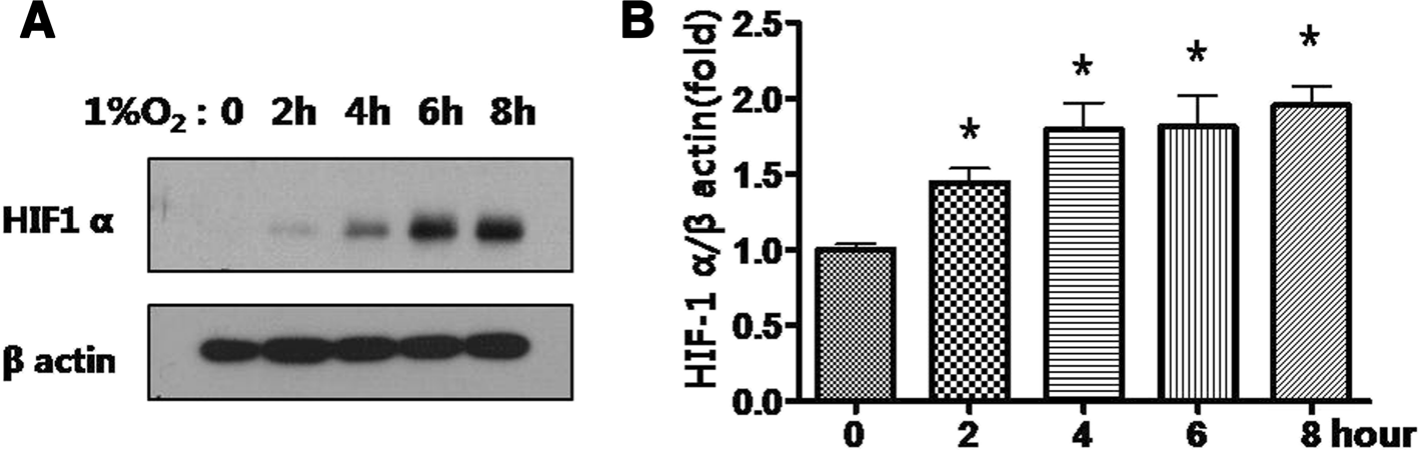
A549 cells exposed to 1 % hypoxia exhibit a time-dependent increase in HIF-1α expression. **a**: Cells were incubated under 1 % hypoxia for 0, 2, 4, 6 or 8 h. Western blots show HIF-1α expression with longer hypoxia durations. **b**: Western blot intensity was measured with Image J. Data are mean ± SEM, *p < 0.05. Graphs represent 7 independent experiments

A549 cells were exposed to 1 % hypoxia for 2, 4, 6 or 8 h, and HIF-1α expression was measured to confirm hypoxia at each time point by Western blot. HIF-1α protein started to increase rapidly within 2 h of exposure to 1 % hypoxia and increased further with longer exposure (Fig. 1).

### Hypoxia induces autophagy in cancer cells

**Fig. 2:**
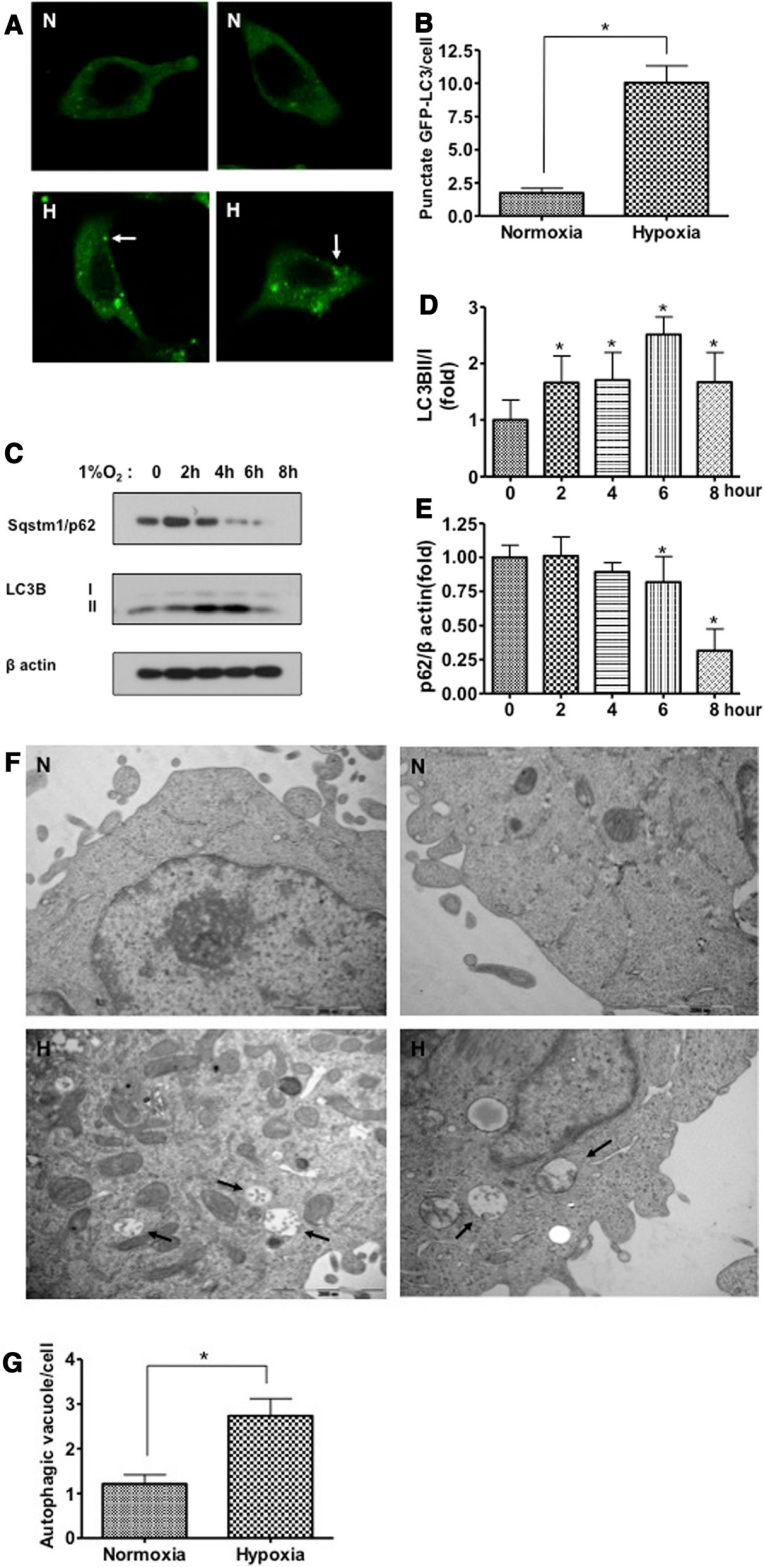
Hypoxia induces autophagy in cancer cells. **a**: LC3-GFP distribution by confocal microscopy. A549 cells were incubated in normoxia or hypoxia for 8 h. GFP-LC3 showed diffuse intracellular localization under normoxic conditions. Membrane translocation (punctate localization) indicative of autophagy was observed in hypoxic cells (magnification x40). White arrows, punctate GFP-LC3. **b**: Quantification of GFP-LC3 translocation in transiently transfected normoxic and hypoxic cells. The number of GFP-LC3 spots per cell was counted. **c**: A549 cells were incubated under normoxia or hypoxia for 2, 4, 6 or 8 h. Western blots showed LC3B-I to LC3B-II conversion in A549 cells incubated in 1 % hypoxia compared with normoxia. Expression of the p62 marker of autophagy flux decreased in A549 cells incubated at 1 % hypoxia compared with normoxia. **d**, **e**: Intensity of Western blots measured by Image J. Graphs represents 6 independent experiments. **f**: Electron micrographs of A549 cell ultrastructure after normoxia or hypoxia for 8 h. Black arrows, double-membraned autophagic vacuoles (magnification x20,000). **g**: Quantification of autophagic vacuoles in normoxic and hypoxic cells. The number of autophagic vacuoles per cell was counted. N, normoxia; H, hypoxia. Data are mean ± SEM, *p < 0.05

Next, we measured autophagy activity under hypoxia. First, we measured expression from a vector encoding green fluorescent protein-tagged microtubule-associated protein 1 light chain 3 (GFP-LC3), which condenses in autophagic vacuoles, resulting in punctate fluorescence within cancer cells undergoing autophagy. A549 cells were transiently transfected with GFP-LC3 plasmids. After 24 h, cells were exposed to normoxia or hypoxia. After 8 h of hypoxia, we counted cells with diffuse or punctate GFP under a confocal microscope. Under normoxia, GFP-LC3 cells were diffusely fluorescent whereas GFP-LC3 cells showed punctate patterns under hypoxia, indicating autophagosome formation (Fig. 2a, b).

Using additional independent assays, we confirmed the involvement of autophagy. We used Western blots to analyze the conversion of LC3B-I to LC3B-II by protein processing, which is a hallmark of autophagy. Levels of endogenous LC3B-I to LC3B-II conversion were substantially increased in A549 cells exposed to 1 % hypoxia compared with cells under normoxia. We also determined levels of p62/sequestosome1 (SQSTM1), the degradation of which is used as an autophagy flux marker. Levels of endogenous p62/SQSTM1 were substantially decreased in A549 cells exposed to 1 % hypoxia compared with cells under normoxia (Fig. 2c, d, e).

Transmission electron microscopy (TEM) was used as another independent assay of autophagy. Cells exposed to hypoxia showed substantial accumulation of autophagosomes with cytoplasmic organelles and other vesicles encapsulated in vacuoles (Fig. 2f, g black arrows), suggesting that hypoxia stimulated autophagy and autophagy was enhanced significantly by hypoxia.

### Hypoxia enhances cancer cell chemo-resistance

**Fig. 3:**
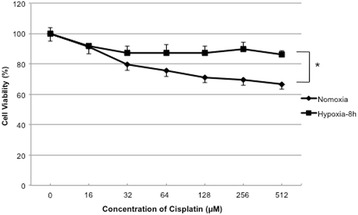
Hypoxia enhances cancer cell chemoresistance. A549 cells were treated with indicated concentrations of cisplatin with normoxia or 1 % hypoxia for 8 h. Cell death was measured by MTT assays. Percent cell death is shown as mean ± SEM of at least 3 independent determinations, *p < 0.05

To investigate the influence of hypoxia on chemo-resistance, A549 cells were exposed to 1 % hypoxia or normoxia for 8 h in the presence or absence of the chemotherapeutic agent cisplatin. Cell death determined by 3-[4,5-dimethylthiazol-2yl]-diphenyltetrazolium bromide (MTT) assays showed that 32.2 % of cells were dead under normoxia and 11.8 % were dead under 1 % hypoxia with cisplatin (512 μМ) (Fig. 3). These data suggested that under hypoxic condition, A549 cells were resistant to a chemotherapeutic agent.

### Blocking autophagy restores sensitivity of cancer cells to chemotherapy

**Fig. 4:**
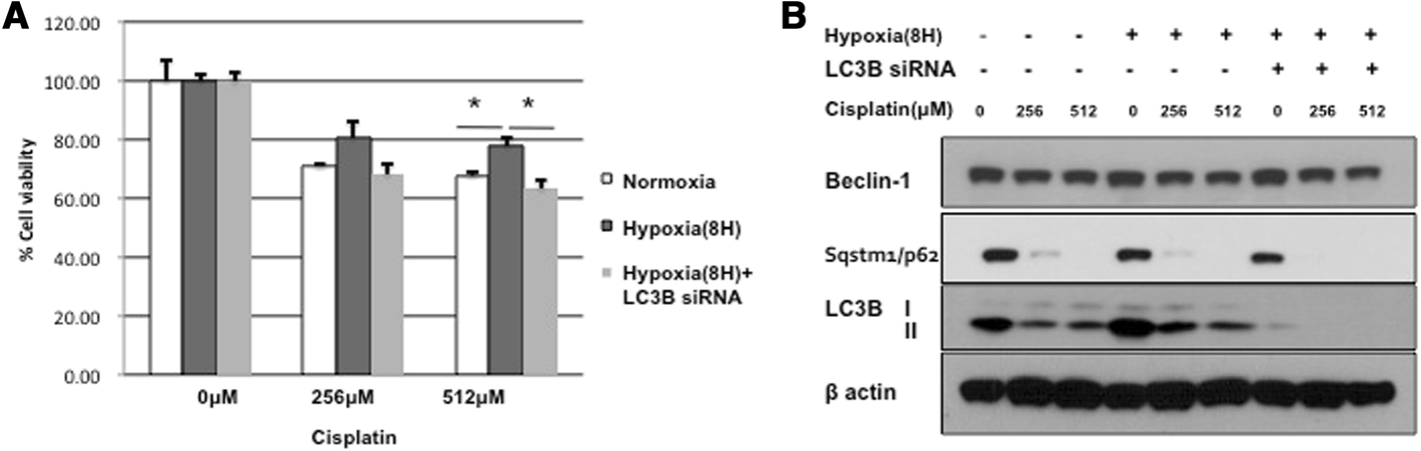
Knockdown of LC3B restored cancer cells sensitivity to cisplatine under hypoxia. **a**: A549 cells were transfected with siRNA negative control or siRNA for LC3B and treated with indicated concentrations of cisplatin under normoxia or hypoxia for 8 h. Cell death was measured by MTT assays. Percent cell death is shown as mean ± SEM of at least 3 independent determinations. **b**: Western blots of LC3B-I to LC3B-II conversion in A549 cells transfected with LC3B siRNA in hypoxia compared with siRNA negative control under normoxia or hypoxia, *p < 0.05

To confirm the involvement of autophagy in cancer cells acquiring resistance to chemotherapy under hypoxia, we inhibited autophagy using LC3B siRNA in cisplatin-treated A549 cells. The siRNA restored the sensitivity of cancer cell to Cisplatin in hypoxia (Fig. 4a). The blocking of autophagy by siRNA was verified by assaying conversion of reduced LC3-I to II (Fig. 4b). These results suggested that activation of autophagy mediated the protective effect of hypoxia on Cisplatin-induced cell death, and siRNA targeting LC3B restored chemotherapy-induced cell death by suppressing autophagy.

### Tumor tissues of lung cancer patients experience hypoxia

**Fig. 5:**
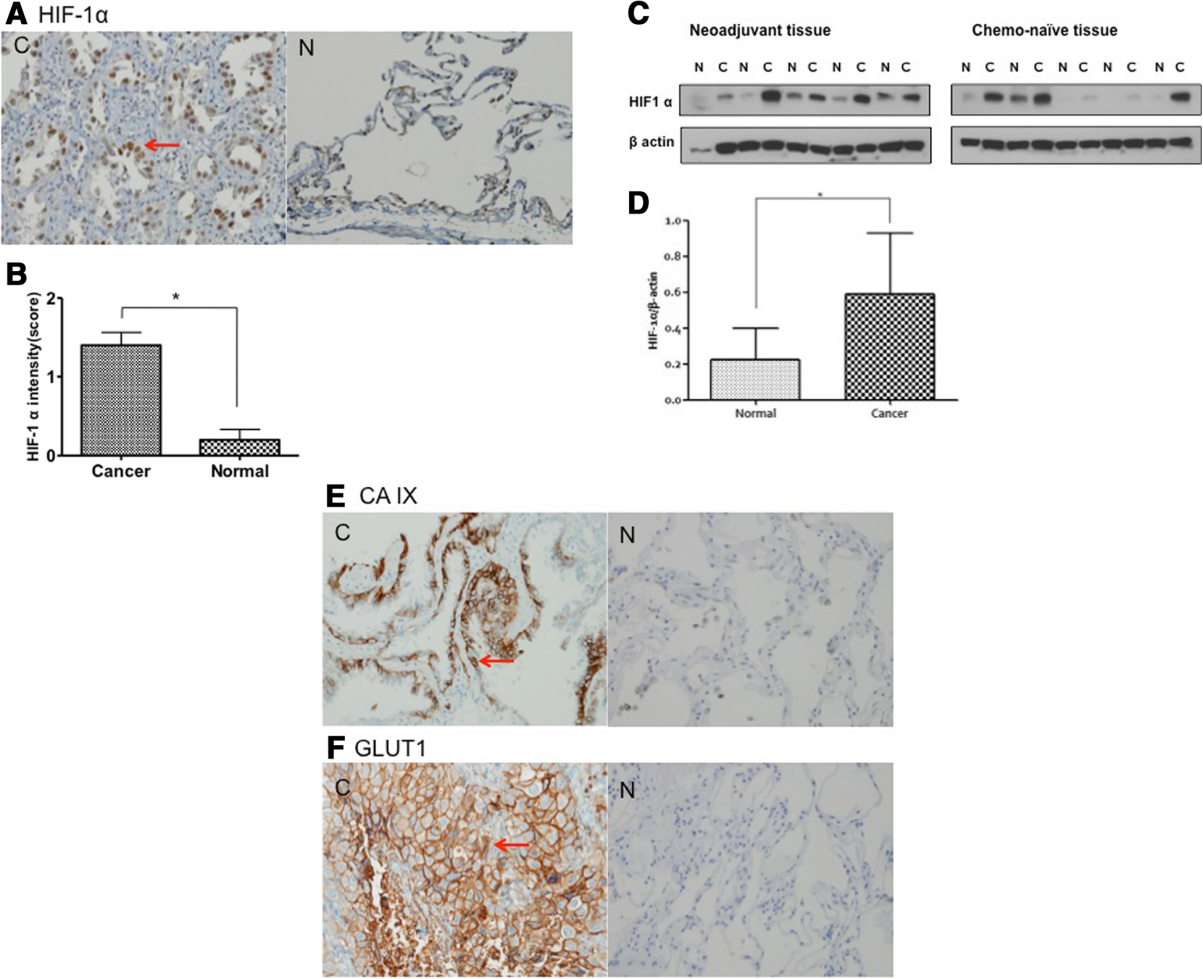
Hypoxia markers of tumor tissues of non-small cell lung cancer (NSCLC). **a**: Immunohistochemical (IHC) stain of tumor tissues shows higher hypoxia inducible factor 1 α (HIF-1α) expression than normal lung tissue. *Red arrow*, HIF-1α expression in tumor cell nucleus (magnification x200). C, cancer; N, normal lung. **b**: HIF-1α staining scored by a semiquantitative grading system based on tumor cells showing completely dark stained nuclei: 0, none; 1, less than 10 % positivity; 2, 10 % or greater positivity. Tissues from ten patients were used for analysis. **c**: Western blots show higher expression of HIF-1α in tumor tissues of NCSLC. Tumor tissues from patients after treatment or with chemonaïve tumors showing HIF-1α expression compared with normal lung tissue. N, normal; C, cancer. **d**: Intensity of Western blots measured using Image J. Tissues from ten patients were used. Data are mean ± SEM, *p < 0.05. **e**. Carbonic anhydrase IX (CA IX) expression by IHC. Five of ten tumor tissues showed positive expression, but no normal tissue showed positive expression (*p* < 0.05). *Red arrow*: CA IX expression (magnification x200). C, cancer; N, normal lung. **f**. Glucose transporter 1 (GLUT1)) expression by IHC. Eight of ten tumor tissues showed positive expression, but no normal tissue showed positive expression (*p* < 0.05). *Red arrow*: GLUT1 expression (magnification x200). C, cancer; N, normal lung

To confirm the effect of hypoxia on solid tumors, we investigated whether hypoxia was seen in tumors of NSCLC patients. HIF-1α expression was assessed by both immunohistochemistry (IHC) and Western blot and compared with normal lung tissue. Tumors showed higher expression of HIF-1-α compared with normal lung tissue on IHC (1.4 vs. 0.2, *p* < 0.05) (Fig. 5a, b). Western blots also showed higher expression of HIF-1α in tumors than in normal lung tissue (Fig. 5c, d).

Besides HIF-1α expression, we also analyzed CA IX and GLUT1 expression by IHC, which are another hypoxia markers, to confirm the hypoxic condition of tumor. All normal tissues showed no expression of both CA IX and GLUT1, but five and eight of ten tumor tissues showed positive expression in CA IX (*p* < 0.05) (Fig. 5e) and GLIT1 (*p* < 0.05) (Fig. 5f) stain respectively. These results indicate that hypoxia is a commonly faced condition in NSCLC tumor.

### Chemotherapy enhanced autophagy induction in tumors of lung cancer patients

**Fig. 6:**
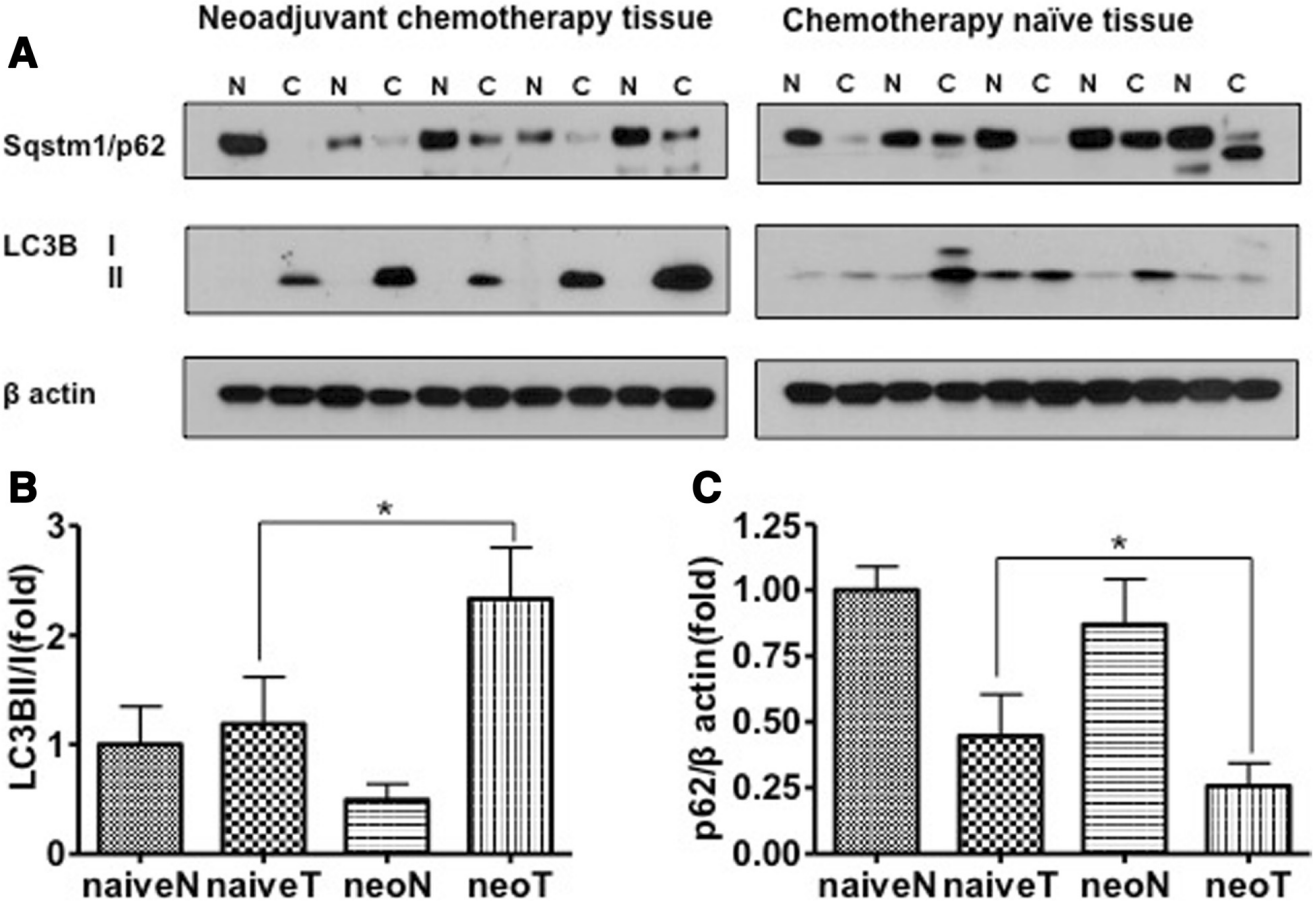
Chemotherapy enhances autophagy in tumor tissues of non-small cell lung cancer (NSCLC). **a** Western blots for conversion of LC3B-I to LC3B-II and p62/SQSTM1 in NSCLC tumors after treatment or in non-treated tumor tissues. Also shown, p62 in tumor tissue after treatment compared to chemotherapy-naïve. **b**, **c**: Intensity of Western blots by Image J. Data are mean ± SEM, *p < 0.05. N, normal; C, cancer; neo, neoadjuvant

To confirm the induction of autophagy in patients with NSCLC after chemotherapy, we analyzed the conversion of LC3B-I to LC3B-II. We also analyzed levels of p62/SQSTM1 in patients with NSCLC who underwent therapeutic pulmonary resection after neoadjuvant chemotherapy, and patients with NSCLC (chemotherapy naïve) who underwent only therapeutic pulmonary resection. Patients who received chemotherapy before pulmonary resection showed increased LC3B-I to LC3B-II conversion and decreased p62/SQSTM1 compared with patients who were chemotherapy naïve (Fig. 6). These results indicated that autophagy was induced by chemotherapy in the tumor tissue of NSCLC patients.

## Discussion

In this study, we found that hypoxia-induced autophagy resulted in enhanced cancer cell survival after chemotherapy. We also confirmed hypoxia and enhanced autophagy activities in response to chemotherapy in human tumor tissues. To our knowledge, this is the first study that demonstrates the role of autophagy especially in hypoxic condition for the developing chemo-resistance in lung cancer.

In spite of recent advances in chemotherapeutic agents, cancer cells show drug resistance that manifests clinically as tumor progression or recurrence. Although many studies have analyzed drug resistance, the mechanisms associated with chemotherapy resistance remain unknown.

Autophagy is important in cellular homeostasis and facilitating cell survival in adverse conditions [15–19] including hypoxia [20–23]. The effect of autophagy on tumors is controversial. Autophagy functions as both a suppressor and a protector of tumor cells [2, 3]. Specifically, after tumor development, autophagy supports the persistence of the tumor. Autophagy is documented in several cancer cells. It is upregulated in response to therapeutic stress caused by chemotherapy and radiation [4–6]. In our study, we showed that autophagy contributes to chemo-resistance in a lung cancer cell line. We also observed enhanced autophagy activity in post-treatment lung cancer tissue after neo-adjuvant treatment. Most advances in understanding autophagy and cancer are from studies using cancer cell lines. Little information is available about autophagy in clinical tumor samples. To investigate our hypothesis, we measured autophagy activity in treatment-naïve tumors and post-treatment tumors from NSCLC patients after neo-adjuvant treatment. This report describes enhanced autophagy activity in human lung cancer tissue after treatment.

Among several conditions that induce autophagy, hypoxia is common microenvironments in solid tumors. Hypoxia is often found in solid tumors including lung cancer and is important because it triggers crucial pathways related cancer progression. Cells adapt to hypoxia through several pathways including metastasis, angiogenesis, autophagy and glycolytic metabolism [24]. Recent studies showed that hypoxia induces autophagy [21–23], and autophagy induced by hypoxia protects tumor cells from apoptosis after chemotherapeutic agent treatment. Aiming the underlying mechanism of hypoxia, the HIF-1α-related pathway is crucial [25–29]. HIF-1α, which is induced by hypoxia, is a main transcription factor and induces autophagy by controlling expression of the downstream targets BNIP3 and BNIP3L [30–32]. We found high expression of HIF-1α in tumor tissues from NSCLC patients, indicating that hypoxia in solid tumors was involved in the tumor microenvironment. In addition, lung cancer cells exposed to hypoxia were resistant to the chemotherapeutic agent.

Autophagy activities in human solid tumor compared to normal tissue have been reported in several tumors. Beclin 1 and LC3 were decreased in human lung cancer but this expression was not associated with gender, smoking, histological type and stage [33] and decreased expression of LC3 was reported in ovarian cancer [34]. However, Yoshioka et al. [35] reported an increased expression of LC3-II in esophageal and gastrointestinal tumors, and Karpathiou et al. [36] reported LC3A as a prognostic factor in lung cancer. In this study, conversion of LC3B-I to LC3B-II couldn’t show any difference between naïve tumor tissues and naïve normal tissues in Fig. 6. Therefore, the expression of autophagy activity itself in several tumors seems not to be clearly understood yet, and it’s surrounding environments such as exposure of chemotherapy or radiotherapy look more important as shown in this study. But further analyses with large number of cases are needed.

This study has some limitations. Only single tumor cell line (A549) and single chemotherapeutic agent (cisplatin) was tested, further experiment with additional cell line and other chemotherapy agents are needed to confirm this finding. On top of that, this study focused on hypoxia induced autophagy activity related to chemo-resistance. However, hypoxia is also considered to be associated with inhibition of DNA repair or cell proliferation that could lead to alter cisplatin sensitivity [37]. Further experiments including several hypoxia related pathway are needed to understand the underline mechanism of this hypoxia related chemo-resistance.

Since hypoxia and autophagy are vital for tumor progression and effective cancer treatments, we predict that drugs that alter the hypoxia-autophagy pathway might be beneficial for improving the responsiveness of chemotherapy in cancer patients. Our study demonstrated that autophagy is pivotal in development of drug resistance in NSCLC, especially in hypoxic conditions.

## Conclusions

Autophagy may play an important role in acquiring resistance to chemotherapy in lung cancer and hypoxia related pathway seems to be involved in autophagy induction.

## Abbreviations

ANOVA: Analysis of variance
BCL2: B-cell lymphoma 2
BNIP3: BCL2/adenovirus E1B 19 kDa interacting protein 3
BNIP3L: BCL2/adenovirus E1B 19 kDa interacting protein 3-like
CA IX: Carbonic anhydrase IX
DNA: Deoxyribonucleic acid
GFP: Green fluorescent protein
GFP-LC3: Green fluorescent protein-tagged LC3
GLUT1: Glucose transporter 1
HIF-1α: Hypoxia-inducible factor 1-alpha
IHC: Immunohistochemistry
LC3: Microtubule-associated protein 1 light chain 3
MTT: 3-[4,5-dimethylthiazol-2yl]-diphenyltetrazolium bromide
mTOR: Mammalian target of Rapamycin
NSCLC: Non-small cell lung cancer
siRNA: Small interfering RNA
RNA: Ribonucleic acid
SEM: Standard error of the mean
SQSTM1: Sequestosome1
TEM: Transmission electron microscope
UPR: Unfolded protein response
